# Supplementary figures and images for: Profiles of Motor-Cognitive Interference in Parkinson’s Disease—The Trail-Walking-Test to Discriminate between Motor Phenotypes
Source: Brain Sci. 2022 Sep 9;12(9):1217. doi: 10.3390/brainsci12091217 (PMC9497145; doi:10.3390/brainsci12091217)

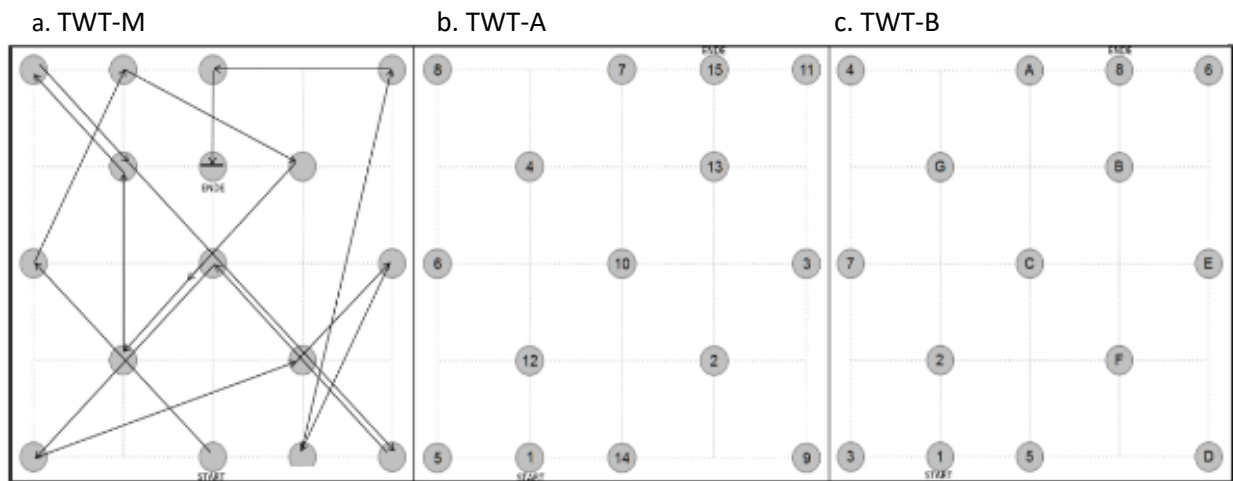

Figure S1: The conditions of the Trail-Walking-Test

Supplement: Supplementary file 1 [file brainsci-12-01217-s001.zip › brainsci-1850141-supplementary.pdf]
